# Assessment of antidiabetic potential and phytochemical profiling of *Rhazya stricta* root extracts

**DOI:** 10.1186/s12906-020-03035-x

**Published:** 2020-09-29

**Authors:** Rashid Mahmood, Waqas Khan Kayani, Tanveer Ahmed, Farnaz Malik, Shahzad Hussain, Muhammad Ashfaq, Hussain Ali, Samina Rubnawaz, Brian D. Green, Danielle Calderwood, Owen Kenny, Gerardo A. Rivera, Bushra Mirza, Faiza Rasheed

**Affiliations:** 1 Department of Biochemistry, Faculty of Biological Sciences, Quaid-i-Azam University, Islamabad, Pakistan; 2 Drugs Control & Traditional Medicines Division, National Institute of Health, Islamabad, Pakistan; 3 Department of Plant Breeding, Swedish University of Agricultural Sciences, Växtskyddsvägen 1, SE-230 53 Alnarp, Uppsala, Sweden; 4 Animal House, National Institute of Health, Islamabad, Pakistan; 5 Institute for Global Food Security, School of Biological Sciences, Queen’s University Belfast, Belfast, Northern Ireland; 6 KTH Royal Institute of Technology, School of Chemical Science and Engineering, SE-100 44 Stockholm, Sweden

**Keywords:** Diabetes mellitus, β-Secretase, DPP-IV, GLP-1 secretion, In vivo studies, Alloxan induced mice, GC-MS

## Abstract

**Background:**

Diabetes mellitus is a chronic disease characterized by hyperglycemia that may occur due to genetic, environmental or lifestyle factors. Natural remedies have been used to treat diabetes since long and many antidiabetic compounds of varied efficacies have been isolated from medicinal plants. *Rhazya stricta* has been used for decades for the treatment of diabetes mellitus and associated ailments. Considering the folkloric use of *R. stricta* against diabetes, it was aimed to investigate the effectiveness of its root extracts against diabetes through in vitro assays and in vivo studies using animal model along with phytochemical profiling through GCMS.

**Methods:**

Various fractions of *Rhazya stricta* obtained through column chromatography were evaluated for a variety of assays including α-glucosidase, Dipeptidyl peptidase-IV (DPP-IV), β-secretase and Glucagon-like peptide-1 (GLP-1) secretion studies. For the in vivo studies the alloxan-induced diabetic mice were treated with root extracts and blood glucose levels, HbA1C, and other biochemical markers along with the histological study of the liver were done. The phytochemical identification was performed using an Agilent 7890B GC coupled to a 7010 Triple Quadrupole (MS/MS) system. GraphPad Prism software version 5.01 was used for statistical analysis.

**Results:**

Majority of the extract fractions showed excellent results against diabetes by inhibiting enzymes DPP-IV (Up to 61%) and β-secretase (Up to 83%) with IC_50s_ 979 μg/ml and 169 μg/ml respectively with increase in the GLP1 secretion. The results of in vivo studies indicated a marked reduction in blood glucose and HbA1c levels along with positive effects on other parameters like lipid profile, liver functions and renal functions of extract-treated mice as compared to control. The histological examination of the liver demonstrated hepatoprotective effects against diabetes led changes and various classes of phytochemicals were also identified through GCMS in different fractions.

**Conclusion:**

The results revealed strong antidiabetic activity of *R. stricta* root with the potential to protect body organs against diabetic changes. Moreover, a variety of phytochemicals has also been identified through GCMS that might be responsible for the antidiabetic potential of *Rhazya stricta* root.

**Graphical abstract:**

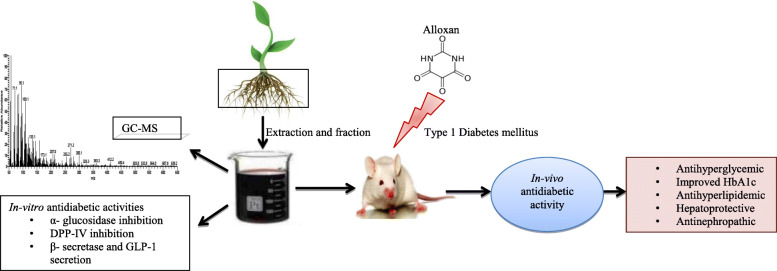

## Background

Diabetes is considered among the top five causes of death in most high-income countries and the half of patients died from diabetes mellitus (DM) were under 60 years of age [1]. The rise in the number of patients of type 2 DM is associated with obesity, hypertension, and an increasingly elderly population and one of the main reasons is the unhealthy lifestyle with no adherence to improved diet and poor exercise [2]. Even though a wide range of therapeutics are available to counter diabetes [3], medical science is still facing a challenge of management of diabetes mellitus with fewer side effects [4].

The use of therapeutic agents and changes in lifestyle are the strategies that can prevent or at least delay the development of DM in patients with impaired glucose tolerance [5]. The use of synthetic drugs for diabetes mellitus leads to secondary complications, therefore; alternative medicines system is taken into account by health-care professionals due to their efficacy, fewer side effects and cost-effectiveness [6]. The antihyperglycemic property of the medicinal plants entirely depends on the chemical constituents present inside the plants [7, 8] and many antidiabetic compounds have been isolated from plants like gelagine, pycnogenol and berberine [9].

*Rhazya stricta* Decne (Apocynaceae) is a folkloric medicinal plant being used in traditional medicine systems against DM [10, 11]. In 1996, Tanira et al. reported an increase in the insulin concentration after administration of *R. stricta* extracts to animals resulting in glucose levels reduction [12]*.* In a study by Ahmed et al., a noteworthy decline in blood glucose levels was observed in diabetic mice after oral administration of extracts fractions of *Rhazya stricta* [13]*.* In another comparative study, Ahmed et al. [14] also reported *Rhazya stricta* as the best in lowering blood glucose levels in animals among other medicinal plants used for the management of diabetes.

Along with other reports, a study by Ali BH [15] reported significant decrease in blood glucose in rats after treatment with the *R. stricta* extracts along with increase in insulin level after 1 h, 2 h and 4 h oral doses of the extracts. Moreover, Baeshen et al. described an improvement in insulin levels in rats along with improved lipid profile and liver function tests by alkaloid rich fraction of *R. stricta* [16]*.*

For DM therapy, the advancement of medical care uses a different array of lifestyle and pharmaceutical interventions [17]. The use of alpha-glucosidase inhibitors like Acarbose, Miglitol, and Voglibose can hinder the release of glucose from complex carbohydrates in the small intestine, consequently suppressing postprandial hyperglycemia. Along with beneficial effects, the use of alpha-glucosidase inhibitors is associated with gastro-intestinal disorders [18]. Likewise, glucagon-like peptide-1 (GLP-1) is an incretin hormone that plays different roles in blood glucose regulation in the gut [19]. This hormone is rapidly inactivated by an enzyme dipeptidyl peptidase-IV (DPP-IV) also present in the gut within less than a minute after being secreted [20]. In the treatment of DM, the inhibition of DPP-IV enzyme is a novel strategy that could potentially affect glucose regulation through multiple effects [21–23]. In addition, the beta-amyloid (APP) cleaving enzyme (BACE1) levels may also play an important role in glucose and lipid homeostasis in conditions of chronic nutrient excess [24] and central BACE-1 level was reported to be higher after insulin deficiency produced through streptozotocin injection [25]. BACE1 is also found to be involved in the regulation of the insulin receptor in the liver [26]. Hence, considering BACE1 as a therapeutic target, efforts to discover and develop BACE1 inhibitors have been pursued in the last few years [27].

To address many biological and biomedical questions and to increase human knowledge, the role of animal models in experimental research cannot be denied. Many of the animal species are being used in the field of biology due to their phylogenetic proximity to humans [28]. Animal models have a major contribution in the field of diabetic research and we can get valuable information about pathways that contribute to the induction of diabetes mellitus in humans [29]. Moreover, animal models are considered as an essential tool to understand the molecular basis, pathogenesis of diabetes and to evaluate the different therapeutic agents [30].

In the present study, keeping in view the folkloric use of *R. stricta* for the treatment of DM, roots of *R. stricta* were evaluated for anti-diabetic effects through various assays including animal study. Alloxan induced diabetic mice were treated with the extracts and effects of root extracts on blood glucose and other biochemical parameters were evaluated. Liver histology of mice was also done to determine the hepatoprotective/ hepatotoxic effects of the extracts on the liver as it is the vital organ involves in the detoxification of metabolites, synthesis of various proteins and other biochemicals necessary for digestion.

## Methods

### Plant collection and extract preparation

Roots of *R. stricta* plant were collected in the month of April from District Karak, KPK, Pakistan, after approval of the study from Bioethics Committee of Faculty of Biological Sciences, Quaid-i-Azam University, Islamabad, Pakistan. Roots were identified by Prof. Dr. Rizwana Aleem Qureshi, Taxonomist, department of plant sciences (Voucher Specimen No. 130290). Roots were carefully inspected and all deteriorated parts or foreign material was separated and thoroughly washed with tap water for the removal of sand or dust particles. After drying, roots were cut into small pieces followed by shade drying for about 2–3 weeks in a well-ventilated area. The dried material was then ground using a laboratory grinder to ease the extraction process.

The crude extracts of dried roots was prepared through maceration (ammonical-chloroform: methanol = 1:1) at room temperature and fractionated through solvent-solvent extraction which resulted in four fractions termed as First Aqueous Layer (FAL), Second Chloroform Layer (SCL), Third Aqueous Layer (TAL) and Forth Chloroform Layer (FCL). In continuation of our previous work [31] in which the Second Chloroform Layer exhibited the most promising results against diabetes; it was decided to further fractionate the SCL fraction through normal phase column chromatography and evaluate for antidiabetic potential.

**Fig. 1:**
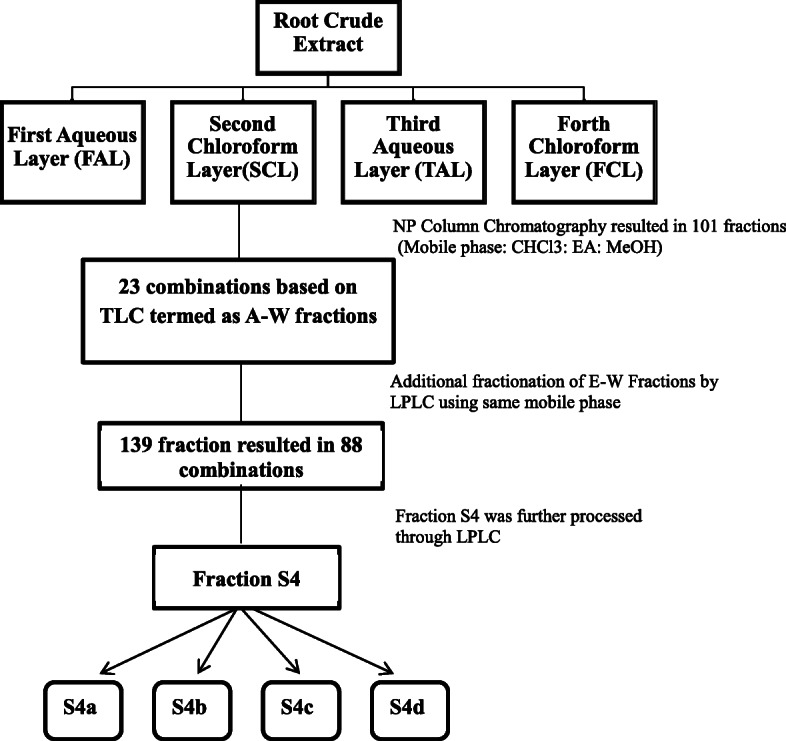
Summery of fractionation of crude extract of *Rhazya stricta* root through normal phase column chromatography

Further fractionation of Second Chloroform Layer (37.3 g) was carried out by normal phase column chromatography using a thick glass column with silica (260–280 mesh) and eluted with chloroform, ethyl acetate, and methanol by gradient change in mobile phase with increasing polarity (starting from 10% ethyl acetate in chloroform to 100% ethyl acetate and then 5% methanol in ethyl acetate to 100% methanol). Similar fractions were combined on the basis of analytical normal phase TLC. Resulting 23 combinations were termed as A-W master fractions and E-W fractions (755-2876 mg) were further purified through flash column chromatography (FCC). The complete fractionation scheme is shown below diagrammatically (Fig. 1).

Fraction S4 was the only fraction obtained as a whitish powdered form containing some noticeable compounds based on TLC which were expected to be isolated easily and weight of fraction was higher than all fractions so it was further processed through column chromatography and eluted with same mobile phases mentioned above and four sub-fractions (S4a, S4b, S4c and S4d) were obtained. Fractions A-D were not further processed due to a minute quantity of waxy material (un-dissolved in the solvent of choice) and in total, 95 fractions (A to W5) were obtained through fractionation of SCL (Table 1). It is important to mention here, that all obtained fractions were tested for the evaluation of antidiabetic potential through in vitro enzyme inhibition assays and on the basis of these results, fractions with good activity were selected for further evaluation as described in the results section of each assay.

**Table 1:** List of fractions obtained from fractionation of Second Chloroform Layer (SCL) processed through column chromatography

Master Fractions	Sub-fractions obtained through column chromatography of Master Fractions with weight (mg)									Total Fractions
**A**	Master Fractions A (321), B (356), C (418), and D (227) were not further fractionated being extremely waxy material.									1
**B**										1
**C**										1
**D**										1
**E**	E1 (234)	E2 (151)								2
**F**	F1 (99)	F2 (224)								2
**G**	G1 (181)	G2 (308)								2
**H**	H1 (157)	H2 (324)	H3 (241)	H4 (196)						4
**I**	I1 (117)	I2 (159)	I3 (236)	I4 (123)	I5 (91)					5
**J**	J1 (44)	J2 (332)	J3 (187)							3
**K**	K1 (78)	K2 (160)	K3 (201)	K4 (155)	K5 (129)					5
**L**	L1 (138)	L2 (364)	L3 (171)	L4 (289)	L5 (83)	L6 (195)	L7 (165)	L8 (99)		8
**M**	M1 (171)	M2 (334)								2
**N**	N1 (224)	N2 (161)	N3 (104)	N4 (87)	N5 (189)	N6 (68)	N7 (181)			7
**O**	O1 (87)	O2 (145)	O3 (133)	O4 (266)	O5 (193)					5
**P**	P1 (143)	P2 (633)	P3 (345)	P4 (188)	P5 (277)	P6 (431)				6
**Q**	Q1 (98)	Q2 (355)	Q3 (289)	Q4 (265)	Q5 (162)					5
**R**	R1 (101)	R2 (266)								2
**S**	S1 (132)	S2 (267)	S3 (191)	S5 (313)	S6 (287)	S7 (411)				6
**S4**	S4a (125)	S4b (89)	S4c (443)	S4d (72)						4
**T**	T1 (79)	T2 (331)	T3 (229)	T4 (279)						4
**U**	U1 (143)	U2 (151)	U3 (198)	U4 (368)	U5 (119)					5
**V**	V1 (57)	V2 (69)	V3 (155)	V4 (389)	V5 (221)	V6 (88)	V7 (101)	V8 (78)	V9 (255)	9
**W**	W1 (221)	W2 (143)	W3 (195)	W4 (277)	W5 (136)					5
								**Total Fractions**		**95**

### Enzyme inhibition assays

#### Dipeptidyl peptidase-IV (DPP-IV) inhibition assay

All obtained fractions were investigated for their inhibitory potential against the dipeptidyl peptidase-IV enzyme as done earlier by Fujiwara & Tsuru [32]. The assessment of DPP-IV inhibition was done fluorometrically using Gly-Pro-aminomethyl coumarin substrate, purified porcine DPP-IV enzyme (1 U/ml) and Berberine (Flouorochem, UK) as a positive control. All samples were run in triplicate using 96 well microtiter plate. Briefly, 20 μl sample, 30 μl AMC substrate and 20 μl DPP-IV enzyme (1 U/ml) were added in each of the respective well and reaction mixture was incubated at 37 °C with gentle agitation for one hour. After incubation, 100 μl Acetic acid (3 mM) was added in each well to stop the reaction. The amount of free AMC after liberation from the substrate is monitored using Excitation and Emission wavelength at 351 nm and 430 nm respectively [33] with the help of the Tecan Safire fluorometer (Reading, England). Extracts were tested at different concentrations like 1.0, 0.5 and 0.25 mg/ml. HEPES buffer was used as negative control and percentage inhibition was calculated by the given formula. Finally, IC_50_ values were calculated which represents the 50% inhibition of DPP-IV activity by each fraction.

$$ \%\mathrm{inhibition}=\frac{\left( Fc- Fs\right)}{Fc}\times 100 $$

Where “Fc” is the fluorescence of negative control and “Fs” is of the sample.

#### α-Glucosidase inhibition assay

Fractions obtained through fractionation of SCL fraction were investigated for α-glucosidase inhibition activity according to the method described earlier [34] with the help of enzymatic assay kit and PGM7 Micro-Stat Analyzer (Analox Instruments Ltd.; London, UK). Briefly, the crude enzyme was prepared from rat intestinal acetone powder (Sigma, UK) with a 50 mM citrate buffer (pH 6.0) in a 1:9 (w/v) ratio and the supernatant obtained was used as the crude enzyme solution for assays. Extract samples were dissolved in PBS buffer pH 7.4 which was prepared by mixing KCl (0.2 g), NaCl (8.0 g), Na_2_HPO_4_ (1.44 g) and KHPO_4_ in 1000 ml of distilled water. Experimentally, the reaction mixture contained substrate (Maltose) 300 μl, rat intestinal acetone extract 10 μl and plant extract 150 μl. Acarbose (Sigma-Aldrich, UK) was used as positive control and PBS buffer as a negative control. The enzyme activity or its inhibition was monitored by the formation of glucose from carbohydrate during the reaction at specific time intervals include 0, 30 and 60 min (the whole intervals) during incubation of reaction mixture at 37 °C using PGM7 analyzer.

#### β-Secretase inhibition assay

β-Secretase or BACE1 activity was done as described earlier by Cox et al., [35] using a kit of Sigma (Sigma-Aldrich, Inc.) with slight modification. The kit was provided with BACE-1 enzyme, substrate, standard, buffer and stop solution. The same buffer was used for the preparation of enzyme and substrate at required concentration 0.3 units/μl and 50 μM respectively according to the manufacturer’s instruction. β**-**secretase inhibitor (KF-14, Mol. Formula C_73_H_118_N_16_O_27_, Mol.wt. 1651.85) was also procured from GL Biochem (Shanghai) Ltd. China. Assay components (test samples/inhibitor/buffer, enzyme, substrate) were mixed to a final volume of 100 μL in each well of a 96-well microtiter plate. The fluorescence level was measured at zero and 2 h after incubation at 37 °C using Excitation and Emission wavelength at 320 nm and 405 nm respectively with the help of Tecan Safire fluorometer (Reading, England, UK). Stop solution was added in all wells to stabilize the signal for up to 24 h.

### Secretion of glucagon-like peptide-1 (GLP-1)

Cell culturing (Secondary cell culture/ Cell lines) and GLP-1 secretion studies were done according to the procedure of Gillespie et al. [36] and measurement of the GLP-1 level using GLP-1 ELISA kit of EMD-Millipore Corporation USA. Dulbecco’s Modified Eagle Medium (DMEM) was used for cell culturing which comprised 4.5 g/l D-glucose with L-glutamine, without sodium pyruvate (Gibco, Paisley, UK) and supplemented with 17.5% fetal bovine serum, 100 U/ml penicillin and 100 μg/ml streptomycin. Culturing was done by seeding about two million cells per well into 12-well plates in 1 ml culture medium with 18 h incubation at 37 °C in a 5% CO2 humidified atmosphere.

Washing with buffer (10 mM Glucose, 20 mM HEPES, 4.5 mM KCl, 140 mM NaCl, 1.2 mM MgCl_2_, 1.2 mM CaCl_2_) was done after removing cells from medium and incubated further for 1 h with the same buffer. The buffer aspired off and cells were incubated for 3 h with 400 μl extract samples. The same volume (400 μl) of HEPES buffer was added in respective well as vehicle (negative) control. In the final step before ELISA, centrifugation of vehicle control and test agents was done to get rid of cellular debris and stored at − 80 °C.

### GLP-1 measurement

Measurement of GLP-1 was done in duplicate by following the procedure of GLP-1 ELISA assay kit of EMD-Millipore, USA. Anti-GLP-1 monoclonal antibodies were already coated in the 96-well microtiter plate. The standards, QC 1, QC 2 and samples were added after assay buffer in respective wells, mixed gently and the plate was incubated at 4 °C for 20–24 h. On next day detection conjugate was added, incubated for 2 h and after washing three times with wash buffer, the substrate was added in each well. After 20 min incubation stop solution was added to stop the reaction and incubated again for a further 5 min at room temperature in the dark to arrest phosphatase activity. The fluorescence was measured using Excitation and Emission wavelength at 355 and 460 nm respectively with the help of the Tecan Safire^2^ fluorometer (Reading, England, UK). The amount of GLP-1 was determined in the sample from the standard curve constructed with standards provided along with the kit. For GLP-1 secretion studies, some selected fractions with good enzyme inhibitory action were evaluated at a single concentration of 1 mg/ml.

### In-vivo studies

On the basis of results of in vitro assays, two fractions S4c and V4 were selected for in vivo studies along with SCL fraction as these fractions showed good inhibitory activity against DPP-IV and BACE1 enzymes along with some GLP-1 secretory potential. Although almost similar results were observed with some other fractions but only S4c and V4 were obtained with reasonable weight, as for animal study a good weight of testing material is required for a group of animals and this was another reason of selection of these fractions. Both these fractions were semi-purified fractions containing off-white powder obtained from fractionation of the second chloroform layer.

### Approval of the study protocol

Study protocols were approved by the Institutional Animal Use and Care Committee (IAUCC) of the National Institute of Health, Islamabad, Pakistan and permission for collection of animal samples was also sought prior to the study. All institutional procedures and protocols regarding animal handling and care (NIH guidelines) were followed and adherence to the ARRIVE guidelines [37] was also taken into account.

### Selection and housing of animals

Briefly, 2 m (8–10 weeks) old healthy male BALB/c albino mice (weight 28.2 ± 1.5 g) were procured from the animal house of the National Institute of Health (NIH), Islamabad, Pakistan. The complete in vivo study was done in the animal house of NIH because of established standardized conditions like 12/12 light-dark cycle with temperature 25 ± 5 °C and relative humidity 35–60%. Initially, all animals were housed in cages to acclimatize for 1 week and fed with standard pellet diet and tap water ad libitum. The cages were solid-bottomed provided with softwood bedding and enough space for movement. Frequent cage cleaning and change of bedding were ensured to maintain hygienic conditions.

### Experimental procedure

Induction of diabetes was done intraperitoneally by injecting freshly prepared 150 mg/kg of alloxan monohydrate (Sigma-Aldrich Co., USA) in normal saline and 5% glucose solution was also placed in bottles to avoid hypoglycemic shock for 24 h. The blood glucose level was checked after 2 weeks of alloxan injection with Glucometer (*ACCU-CHEK*® Performa, Roche) by tail puncture and mice with fasting glucose level > 200 mg/dl were selected for the study. Fifty-four mice were randomly grouped into nine groups each containing six mice as described below and every group was housed in a separate cage with free access to pellet diet and water.

- Group I- Normal Control (NC)
- Group II- Alloxan-induced Diabetic Untreated Control (DC)
- Group III- Alloxan-induced Diabetic Treated Control received Glibenclamide 5 mg/kg b.wt/day (TC)
- Group IV- Alloxan-induced Diabetic received SCL extract 10 mg/kg b.wt /day (SCL-I)
- Group V- Alloxan-induced Diabetic received SCL extract 20 mg/kg b.wt /day (SCL-II)
- Group VI- Alloxan-induced Diabetic received S4c fraction 10 mg/kg b.wt /day (S4c-I)
- Group VII- Alloxan-induced Diabetic received S4c fraction 20 mg/kg b.wt /day (S4c-II)
- Group VIII- Alloxan-induced Diabetic received V4 fraction 10 mg/kg b.wt /day (V4-I)
- Group X- Alloxan-induced Diabetic received V4 fraction 20 mg/kg b.wt /day (V4-II)

All samples were given through oral intubation in 5% DMSO in physiological saline [38] and to harmonize the experiment, 5% DMSO in saline was also given to normal and diabetic untreated control groups throughout the study.

### Blood collection and samples processing

On 28th day at 9:00 am, all animals were anesthetized {Forane® (Isoflurane) Batch No. 6048245 of Abbott Laboratories (Pakistan) Ltd.}without fasting. Anesthesia was given by expert veterinarian using cotton soaked with 3% Isoflurane individually by drop jar method aiming to immediate effects and less distress. Blood was withdrawn through heart puncture (diaphragmatic approach) and placed in proper blood collection tubes. Euthanasia was carried out immediately after each blood collection by higher doses of anesthetic. Animals were given few more minutes in the chamber and death was confirmed by examining absence of respiratory activities, no heartbeat and no response to toe pinch. After that, livers were dissected and preserved in 10% formalin for histopathology.

After complete tissue processing of liver [39], microscopic examination was done using 40x objectives (Nikon). All biochemical parameters like blood glucose level, glycosylated hemoglobin (HbA1C), alanine transaminase (ALT), aspartate transaminase (AST), alkaline phosphatase, urea, creatinine, uric acid, cholesterol, triglyceride, total protein, and albumin were measured with the help of fully automated Spectrophotometer “Vitalab Selectra E” of Vital Scientific N.V. /Clinical Data Inc. USA, using reagents of “*DiaSys”* Diagnostic Systems, GmbH Germany.

### GC-MS analysis

The GC-MS analysis was performed using an Agilent 7890B GC with the split-splitless inlet, coupled to a 7010 Triple Quadrupole (MS/MS) system from Agilent, equipped with EI ion source. The system was operated by MassHunter Software with MSD ChemStation. The separation was carried out on an Agilent J&W DB-5 ms capillary column (30 m × 0.25 mm i.d., 0.25 mm film thickness). Helium (purity 99.999%) was employed as carrier gas at a constant column flow of 1.0 mL/min. The GC oven temperature was programmed from 50 °C (held 1 min) to 270 °C at 10 °C/min (held 5 min). The temperature of the transfer line, the quadrupole and the ion source were set at 280, 150 and 230 °C respectively. The injector temperature was set at 250 °C. The injector was operating in the split-less mode and programmed to return to the split mode after 2 min from the beginning of a run. The MSD was operated in full scan acquisition mode. Mass spectra deconvolution of chromatographic signals and tentative identification of unknown bioactive compounds was carried out using the Agilent MassHunter Unknown Analysis tool and the NIST Mass Spectral Library (NIST MS Search 2.0).

### Statistical analysis

Statistical analysis was performed using GraphPad Prism software version 5.01. All values are expressed as mean ± SEM using one-way ANOVA with Tukey’s posttest for comparison. The *p*-value ≤0.05 was considered significant.

## Results

All of the samples in the vehicle were given to mice for 4 weeks until sacrifice and no restlessness, any irritation or any adverse event was noted during the study.

### DPP-IV inhibition

**Fig. 2:**
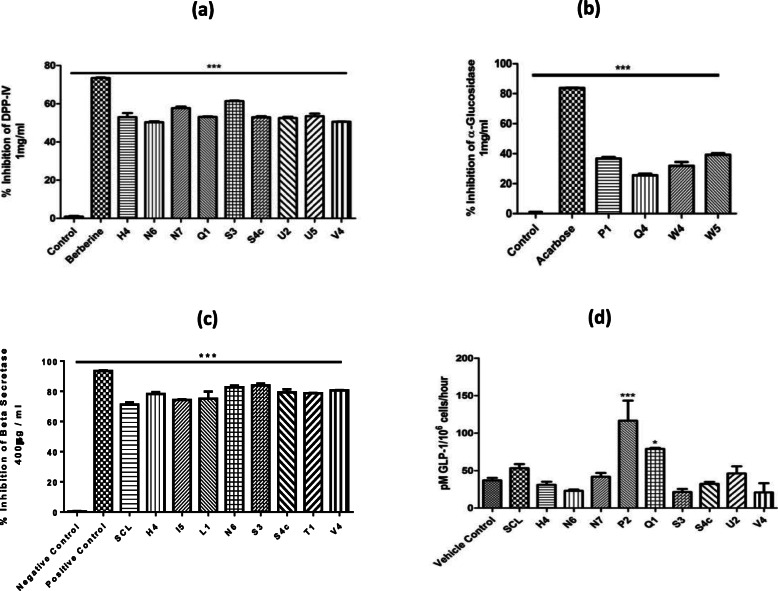
Enzymes inhibition, DPP-IV **a**, α-glucosidase **b**, β-secretase **c** and GLP-1 secretory activity **d** of some selected fractions. Values are expressed as mean ± SEM. (*p < 0.05, ***p < 0.001)

The fractions obtained from the second chloroform layer have displayed significant activity against diabetes by inhibiting DPP-IV enzyme (*p* < 0.001). Initially, all fractions obtained from the second chloroform layer (SCL) were analyzed at a single concentration (1 mg/ml) and then some fractions were chosen on the basis of the highest DPP-IV inhibition and processed further. The majority of fractions showed a good dose-dependent inhibitory response to DPP-IV but some of them like H4, N6, N7, Q1, S3, S4c, U2, U5, and V4, have displayed marked inhibition of DPP-IV (Fig. 2a). IC_50_ values are therefore calculated using a linear regressing curve with interpolation from the standard curve and given in Table 2.

**Table 2:** IC_50_ values of DPP-IV inhibition by root extract fractions of *R. stricta*

Fraction	IC_**50**_ (μg/ml)	Fraction	IC_**50**_ (μg/ml)
**H4**	1440.845	S4c	979.1997
**N6**	1822.220	U2	1280.141
**N7**	1115.541	U5	1359.150
**Q1**	1279.503	V4	1550.559
**S3**	1169.485	Berberine (Positive Control)	35.3

### α-Glucosidase inhibition assay

Alpha-glucosidase inhibition assay was performed for all fractions of the second chloroform layer (SCL) initially at 1 mg/ml concentration to pick fractions with the highest inhibition. Most of the fractions were able to inhibit the enzyme activity but fractions P1, Q4, W4, and W5 exhibited maximum (> 25%) inhibition activity like 36.7, 25.5, 31.8 and 39.2% respectively (Fig. 2b). Three fractions with the highest activity were further processed for the calculation of IC_50_ value which caused enzyme inhibition in a concentration-dependent manner. IC_50_ values are shown in Table 3.

**Table 3:** IC_50_ values of α-glucosidase inhibition by root extract fractions of *R. stricta*

Fraction	IC_**50**_ (μg/ml)
P1	4373
W4	4625
W5	4652
Acarbose	51.3

### β-Secretase inhibition

The second chloroform layer and its fractions represented promising inhibitory effects against the BACE-1 enzyme. Initially, all fractions were analyzed at 400 μg/ml concentrations and based on these results, only fractions with > 70% BACE-1 inhibitory activity (Fig. 2c) were re-examined at a concentration ranging from 50 μg/ml to 400 μg/ml to determine the IC_50_ values_._ The IC_50_ values for the highly active fractions are afforded in Table 4. All fractions showed inhibition in a dose-dependent manner.

**Table 4:** IC_50_ values of some fraction with good β-secretase inhibitory activity

Fractions	IC_**50**_ (μg/ml)	Fractions	IC_**50**_ (μg/ml)
**SCL**	169.26	**S3**	209.33
**H4**	215.30	**S4c**	211.34
**I5**	234.38	**T1**	263.27
**L1**	265.74	**V4**	212.37
**N6**	216.18	**Positive Control**	38.4

### Secretion of GLP-1

GLP-1 secretory studies are made to examine the secretory response of some of the selected fractions in cultured cells at a single concentration (1 mg/ml) and measurement was done with ELISA based kit. The fraction P2 showed a significant increase in acute GLP-1 secretion by 116.6 pM/106 cells (*p* < 0.001) while fraction Q1 also increased secretion by 79.0 pM/106 cells (*p* < 0.005). All other tested fractions were unable to stimulate a significant response (*p* > 0.05). Overall, the fraction P2 was able to increase GLP-1 secretion by 3.3 fold (p < 0.001) while fraction Q1 exhibited a good response by 2.2 fold increase (*p* < 0.05) as compared to vehicle control (Fig. 2d).

### In vivo studies

#### Reduction of blood glucose and glycosylated hemoglobin levels

**Fig. 3:**
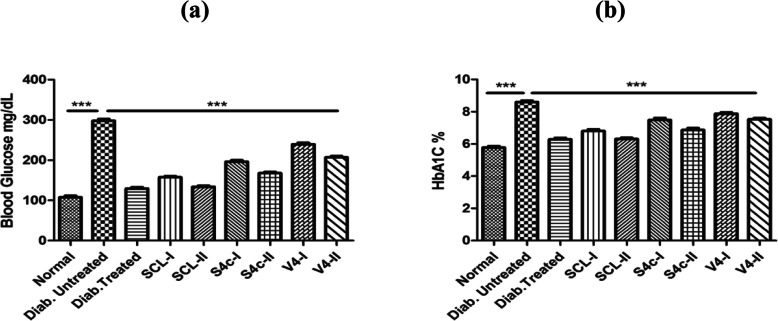
Blood glucose levels **a** and glycosylated hemoglobin **b** determined in all groups of mice after treatment. Values are expressed as mean ± SEM (*p < 0.05, **p < 0.01, ***p < 0.001)

A significant increase in glucose concentration was observed in mice of the untreated diabetic control group (DC) as compared to the normal control group (NC). After 28 days of oral administration of the extract fractions, an observable reduction was noted in the glucose level of almost all groups in comparison with the untreated control group (Fig. 3). Similar results were observed in glycosylated hemoglobin (HbA1C) level and the noticeable decrease was shown by all treated groups of mice as compared to the untreated diabetic control group (DC). Overall, maximum activity has been shown by the SCL-II and its glucose-lowering effects are comparable with the results of the group treated with standard drug glibenclamide (TC).

#### Anti-hyperlipidemic potential of *R. stricta* root

**Fig. 4:**
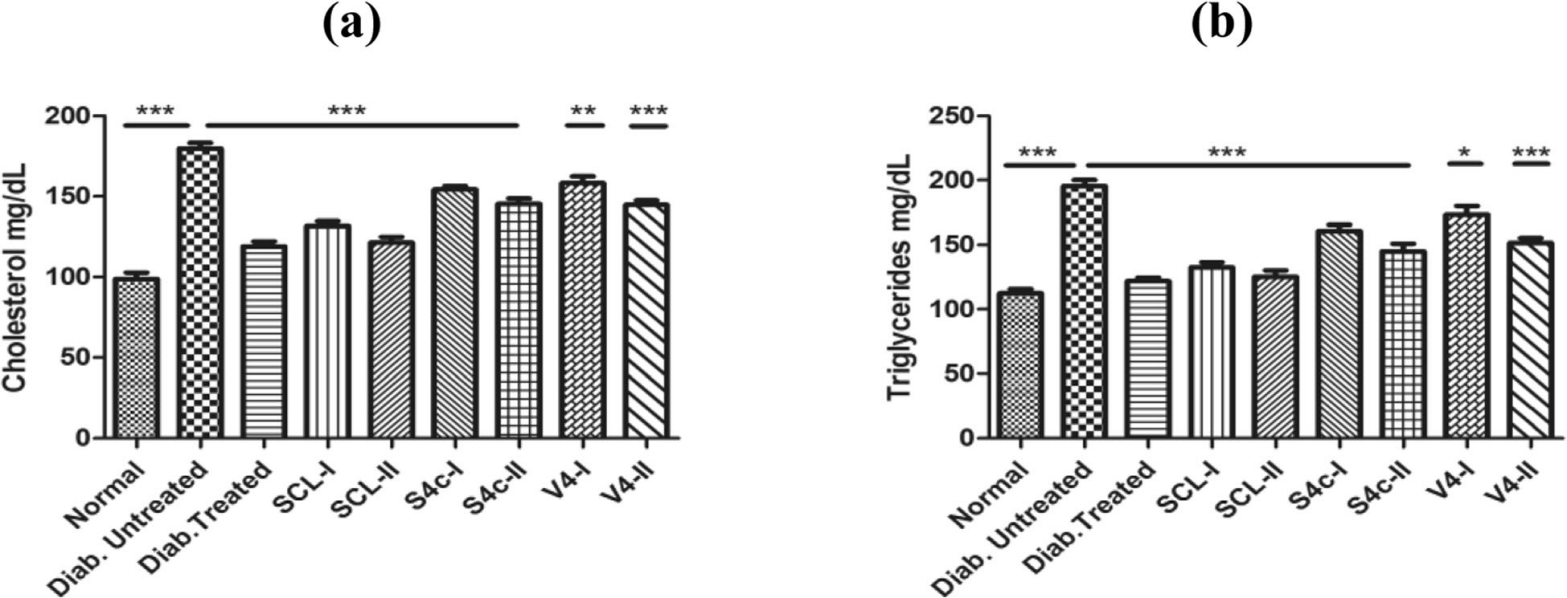
Results of cholesterol **a** and triglycerides **b** determined in all groups of mice after treatment. Values are expressed as mean ± SEM (*p < 0.05, **p < 0.01, ***p < 0.001)

The mice of untreated diabetic control groups (DC) have shown an increase in cholesterol and triglycerides level as compared to the normal control group (NC). On the other hand, a noteworthy decrease in these parameters was observed in the mice of the extract-treated group including the diabetic treated control group (TC) as compared to the untreated diabetic control group (DC). However, the best results have been observed in mice of the group treated with fraction SCL-II (Fig. 4).

#### Effects of root extract on kidney functions tests

**Fig. 5:**
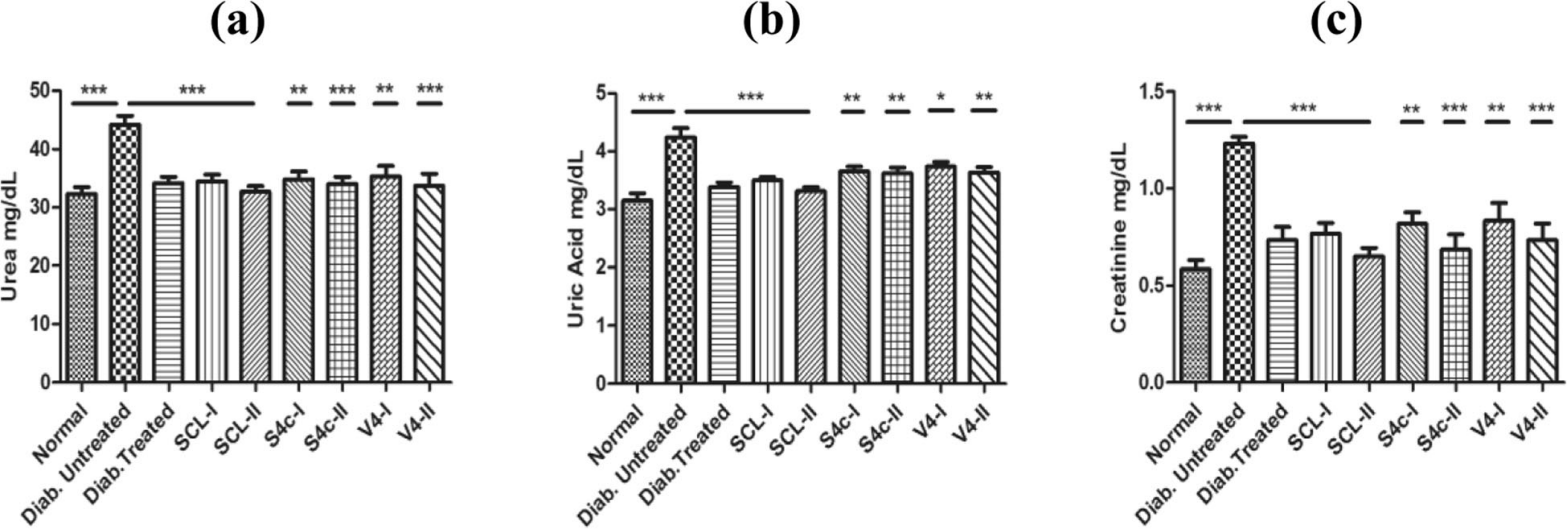
Results of urea **a**, uric acid **b** and creatinine **c** obtained in the blood of mice of all treated groups expressed as mean ± SEM (*p < 0.05, **p < 0.01, ***p < 0.001)

It has been observed that the root extract fractions showed a positive effect on kidney functions of the mice of extract-treated groups. All parameters (urea, creatinine, uric acid) were observed to be high in the diabetic untreated control group and treatment of the mice with the extract fractions decreased blood urea, creatinine and uric acid levels in all groups on comparison with the diabetic untreated control group (Fig. 5).

#### Liver profile and the histological study

**Fig. 6:**
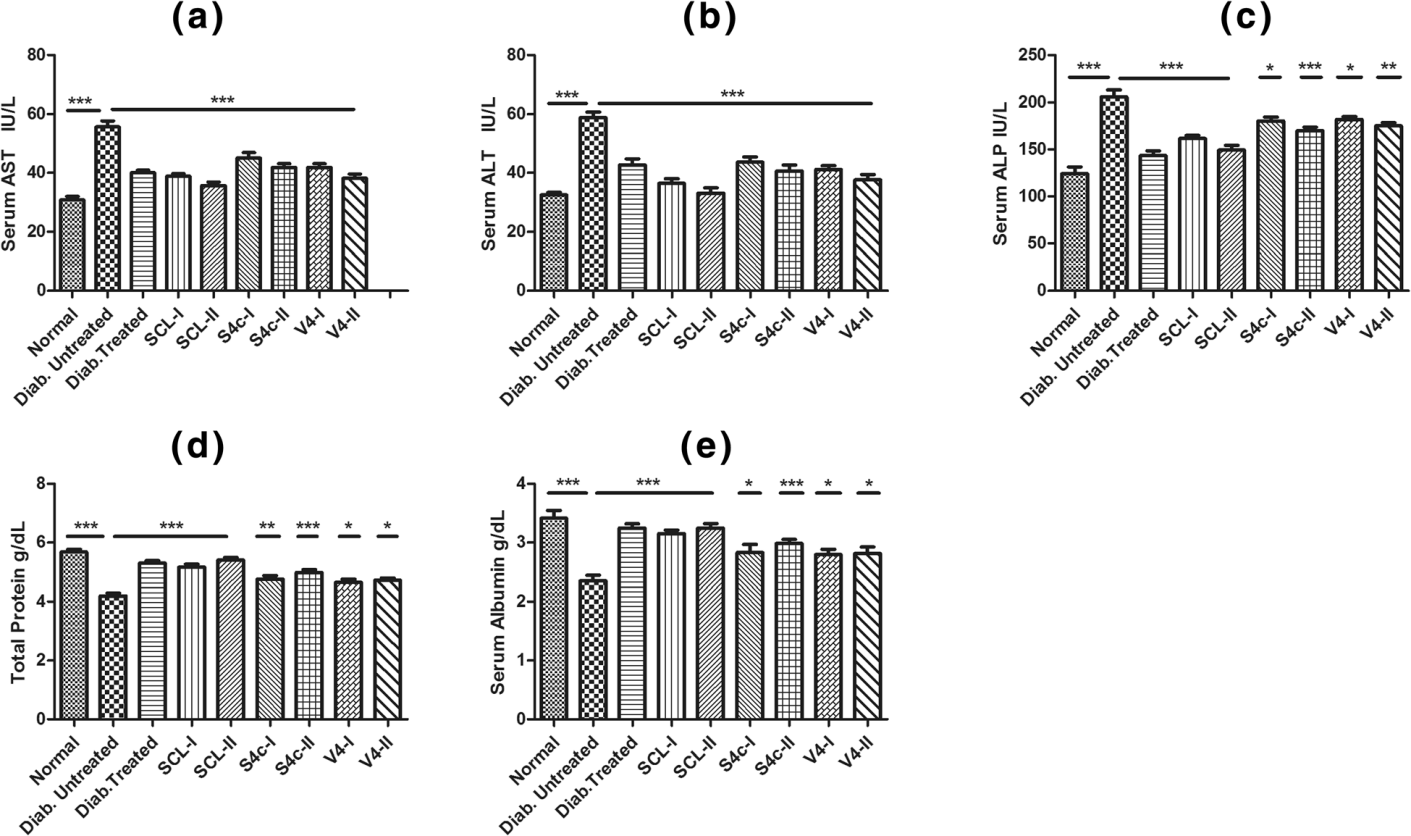
Effects of different extract fractions on the liver profile including AST **a**, ALT **b**, ALP **c**, total protein **d** and albumin **e**. Results are expressed as mean ± SEM (*p < 0.05, **p < 0.01, ***p < 0.001)

The liver profile done during this study has shown strong hepatoprotective effects of the root extract of the *R. stricta* against diabetic changes. Liver enzymes of mice of the diabetic untreated diabetic control group (DC) were increased while a marked decrease was observed in the total protein and albumin contents of mice of this untreated control group as compared to the normal control group (NC). The root extract and its fractions exhibited strong hepatoprotective effects as a significant decrease (*p <* 0.001) was observed in the liver enzymes like AST, ALT and ALP along with improvement in total protein and serum albumin levels in the extract-treated groups as compared to the diabetic untreated control group (DC) (Fig. 6).

**Fig. 7:**
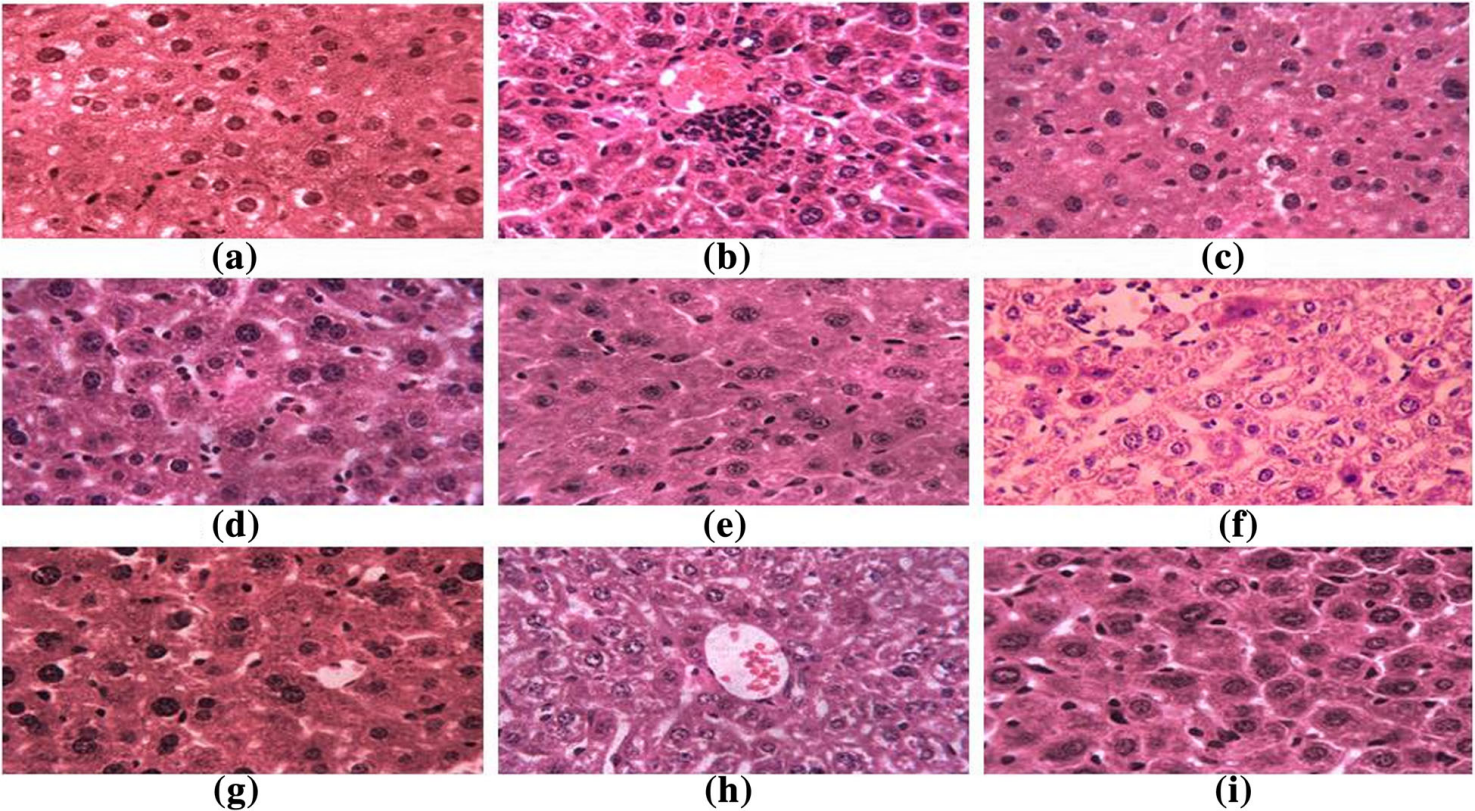
Representative microscopic photographs of liver sections of mice of all groups (H&E Staining): Liver sections of normal mice showing normal hepatic cells **a**, untreated diabetic mice showing vascular dilation, congestion and infiltration of inflammatory cells **b**, treated mice with standard drug **c**, treated with fraction SCL-I **d**, treated with fraction SCL-II **e**, treated with fraction S4c-I **f**, treated with fraction S4c-II **g**, treated with fraction V4-I **h** and liver section of diabetic mice treated with fraction V4-II **i**

Histological examinations of liver sections of the normal control group showed normal hepatocytes and veins with uniformity in cells but on the comparison, the untreated diabetic control group (DC) was observed to have hepatic alterations like vascular dilation, congestion and increased number of inflammatory cells. The extract fractions presented their hepatoprotective effects and preserved the normal texture of liver cells and no major cellular alterations were noted in the treated mice groups (Fig. 7).

#### Identification analysis by GC-MS

The results obtained from the GC-MS analysis led to the tentative identification of a good number of compounds, based on the positive match of experimental mass spectra with NIST MS database, and data reported in the literature. The fractions showed the presence of many classes of compounds like diterpenes, sesquiterpenes, phenolics, quinolines, quinoxaline, isoxazoles, phenyl indoles, carbonyls, beta-carboline, and other indole group-containing compounds. Many phytochemicals were identified in the SCL fraction including alkaloids such as aspidospermidine, eburnamenine, quebrachamine, aspidofractinine, eburenine (1,2-didehydro-aspidospermidine), β-hydroxyquebrachamine, secoaspidospermidine as well as several fatty acid methyl esters like heptadecanoic acid methyl ester, 9-octadecenoic acid (Z)- methyl ester and eicosanoic acid methyl ester along with identification of several other heterocyclic compounds such as 2,4-dimethyl-quinoline, hexahydro-1H-Isoindole-1,3(2H)-dione and 4-(3-methyl-2-butenyl)-1H-Indole. Moreover, some Vinca alkaloids have also been identified for the first time in *Rhazya stricta* root like Vinburnine, Vincamine and Apovincamine.

Many of the compounds present in the SCL fraction were also identified in other fractions like aspidospermidine, 1,2-didehydroaspidospermidine, quebrachamine and some fatty acid methyl esters. The other analyzed fractions exhibited many compounds similar to the main fraction SCL and various other compounds which have not been identified in the main fraction SCL like N-methyl-Aspidodasycarpine, Talbotine, Astaxanthin, p-Cymene, Nerinine, 2-Anthracenamine, alfa-Copaene, Deoxyspergualin, D-Limonene and dozens of other compounds with pharmacological potential. A complete list of phytochemicals identified in fraction SCL with their presence in all other fractions is given in Table 5.

**Table 5:** List of compounds identified in the Second Chloroform Layer (SCL) fraction and their presence (P) in other fractions obtained from SCL

RT	Compounds identified in SCL fraction	Fractions obtained from SCL											
		H4	N6	N7	P1	Q1	S3	S4c	U2	V4	W5	Synonyms (PubChem CID)	Reported Antidiabetic/ Antioxidant Activity
**4.039**	2H-Pyran, tetrahydro-2-(12-pentadecynyloxy)-								P				
**5.441**	Azocine, octahydro-	P				P	P						
**5 .802**	8-Azabicyclo[3.2.1]oct-2-ene	P			P		P		P				
**5.866**	2-Amino-6-methoxypyridine												
**5.996**	4(1H)-Pyridinone, 2,3-dihydro-1-methyl-							P					
**6.271**	Tetratetracontane												
**7.263**	Pyridine, 3-ethyl-5-methyl-							P		P			
**7.346**	1,3-Propanediol, 2-butyl-2-ethyl-									P			
**7.665**	Nonane, 4,5-dimethyl-	P	P	P			P						
**9.095**	Hexadecane	P	P			P	P		P	P			
**9.736**	Dodecane, 4,6-dimethyl-	P	P	P		P	P						
**10.238**	Dodecane, 1-iodo-		P	P		P	P						
**10.658**	1-(3-Aminopropyl)-2-pipecoline	P	P	P		P	P		P	P	P		
**10.720**	2-Isopropyl-5-methyl-1-heptanol	P	P	P		P	P						
**10.864**	Dodecane, 1-iodo-	P	P	P		P	P						
**10.990**	Dodecane, 2,7,10-trimethyl-	P		P			P						
**11.08**	4,4′-Isopropylidenebis (3-methyl-2-isoxazolin-5-one)							P		P			
**11.360**	Cyclohexanamine, N-methyl-n-propyl-	P	P		P	P	P	P	P	P			
**11.596**	Acetamide, 2-(3-hydroxy-8-aza-bicyclo [3.2.1]oct-8-yl)-N-(2,4,6-trimethylphenyl)-	P				P	P	P					
**11.874**	1H-Isoindole-1,3(2H)-dione, hexahydro-									P			
**12.332**	*n*-Hexadecane		P				P						Inhibition of enzymes involved in glucose metabolism [40]
**12.641**	Heptacosane	P		P		P	P						
**12.871**	Decane, 2,3,5,8-tetramethyl-							P					
**12.981**	Quinoline, 2,4-dimethyl-				P					P			
**13.063**	Eicosane, 1-iodo-	P		P						P			
**13.178**	Heptacosane	P		P			P						
**13.293**	2,4-Di-tert-butylphenol	P	P		P	P	P	P		P	P		
**13.345**	((8R,8aS)-8-Isopropyl-5-methyl-3,4,6,7, 8,8a-hexahydronaphthalen-2-yl)-methanol												
**13.411**	11-Methyldodecanol	P			P			P					
**13.528**	1-Decanol, 2-hexyl-					P							
**13.606**	Heptacosane	P	P			P	P						
**13.737**	Cyclohexane, 1-ethyl-2-propyl-	P					P						
**13.911**	1-Naphthalenamine, N-ethyl-				P					P	P		
**13.961**	3′,5′-Dimethoxyacetophenone					P							
**14.041**	Guanethidine	P			P				P				
**14.714**	Heptadecane, 2,6,10,15-tetramethyl-												
**14.134**	Pyridine, 3-methyl-4-phenyl-							P		P			
**14.287**	Heptacosane							P					
**15.119**	Humulene												
**15.212**	Heptacosane	P	P			P	P						
**15.278**	Heptadecane, 2,6,10,15-tetramethyl-		P			P	P						
**15.560**	2-Bromotetradecane						P						
**15.682**	1-Naphthalenamine, N-ethyl-												
**15.887**	2-Tetradecanol												
**15.980**	Carbonic acid, eicosyl vinyl ester	P				P							
**16.028**	Dodecane, 1-iodo-	P				P							
**16.127**	Heptacosane												
**16.185**	1,7-Dimethyl-4-(1-methylethyl)cyclodecane	P		P									
**16.280**	Methoxyacetic acid, 2-pentadecyl ester												
**16.487**	Thiourea,(5,5-dimethyl-3-oxo-5,6-dihydropyrrolo[2,1-a]isoquinolin-2-ylidene)-						P						
**16.540**	11-Methyldodecanol	P											
**16.852**	Sydnone, 3-(2-naphthyl)-												
**16.958**	Ethanone, 2-chloro-1H-indol-1-yl-	P		P			P						
**17.028**	2-Propenoic acid, 3-(4-hydroxy-3-methoxyphenyl)-, methyl ester		P	P									
**17.139**	1H-Indole, 4-(3-methyl-2-butenyl)-												
**17.288**	1,2-Benzenedicarboxylic acid, bis(2-ethylpropyl) ester							P		P			
**17.458**	Eicosane, 1-iodo-												
**17.547**	Pyrrolidine-2,5-dione, 1-(3-chlorophenyl)-3-(4-phenyl-3,6-dihydro-2H-pyridin-1-yl)-	P	P	P	P	P	P	P	P		P		
**17.584**	(6-Methylquinolin-2-yl)methanamine			P	P								
**17.714**	Heptacosane	P		P		P	P						
**17.797**	Hexadecanoic acid, methyl ester				P								
**17.833**	1,7-Dimethylene-2,3-dimethylindole												
**18.032**	Benzenepropanoic acid, 3,5-bis(1,1-dimethylethyl)-4-hydroxy-, methyl ester			P		P	P						
**18.096**	Isobutyl tetradecyl ether												
**18.156**	n-Hexadecanoic acid	P	P	P		P	P						Antidiabetic [41]
**18.211**	Eicosane, 1-iodo-					P	P						
**18.370**	Hexadecane			P									
**18.666**	Carbonic acid, eicosyl vinyl ester	P		P			P						
**19.479**	trans-13-Octadecenoic acid, methyl ester		P								P		Antioxidant [42]
**19.526**	9-Octadecenoic acid (Z)-, methyl ester	P	P	P	P	P	P	P	P				Antioxidant [42]
**19.630**	Hexadecane						P						
**19.699**	Methyl stearate	P		P	P	P							
**19.839**	9,12-Octadecadienoic acid (Z,Z)-		P	P	P	P			P				
**19.862**	3-Amino-5-chloro-benzofuran-2-carboxylic acid methyl ester												
**20.443**	Heptacosane, 1-chloro-					P	P	P		P			
**20.502**	o-(2,4-Dinitrostyryl)-phenol		P										
**20.932**	Aspidofractinine	P					P						
**20.986**	Aspidospermidine, 1,2-didehydro-, (5.alpha., 12.beta.,19.alpha.)-		P	P	P	P		P		P	P		
**21.053**	Carbonic acid, octadecyl vinyl ester	P	P			P	P				P		
**21.193**	Heptadecane, 8-methyl-												
**21.445**	Eicosanoic acid, methyl ester				P								
**21.662**	Heptadecane, 8-methyl-												
**21.722**	Octadecane, 3-ethyl-5-(2-ethylbutyl)-	P	P	P		P	P	P		P	P		
**21.779**	Oxiranedodecanoic acid, 3-octyl-, cis-												
**21.891**	Fumaric acid, monoamide, N,N-dimethyl-, 1-naphthyl ester												
**22.039**	.beta.-Hydroxyquebrachamine	P			P	P	P						
**22.053**	Hexanedioic acid, bis(2-ethylhexyl) ester		P	P	P	P	P	P	P	P			
**22.157**	Chromium, (1-methoxy-3,3-diphenylpropylidene) pentacarbonyl-												
**22.317**	5,8-Dimethylquinoxaline												
**22.335**	Acetic acid, 6-morpholin-4-yl-9-oxobicyclo[3.3.1]non-3-yl ester										P		
**22.483**	1,2,5-Oxadiazole-3-carboxamide, 4-amino-N-[2-[[(3-chlorophenyl) methyl] amino] ethyl]-				P				P				
**22.548**	Naphtho[1,2-b]furane-2,8-dione, 2,3,3a,4,5,5a,8,9b-octahydro-9-methyl-3-(3,3-dimethyl-1-piperidylmethyl)-	P		P	P	P	P	P	P	P			
**22.595**	(.+/−.)-Uleine												Antioxidant [43]
**22.664**	Apparicine, Nb-methyltetrahydro-												
**22.686**	octadecanoic acid, 3-oxo-, ethyl ester												
**22.843**	1H-Cyclopent[ij]indolo[2,3-a] quinolizine,13a-ethyl-2,3,5,6,6a,11,12, 13,13a,13b-decahydro-11-methyl-, [6aR-(6a.alpha.,11aS*,13a.beta.,13b. beta.)]-											Vallesamidine (579878)	
**22.859**	2,20-Cyclo-8,9-secoaspidospermidine, 3-methyl-, (2.alpha., 3.beta.,5.alpha., 12. beta., 19.alpha.,20R)-												
**22.894**	1-Methyl-16-methoxyaspidospermidin-4-one	P											
**22.997**	12H-benzo[b]phenoxazine, 12-methyl-												
**23.061**	Methyl 8-methyl-nonanoate	P			P	P			P				
**23.169**	1H-Indolo[3,2,1-de]pyrido[3,2,1-ij][1,5]naphthyridine, 13a-ethyl-2,3,5,6,13a,13b-hexahydro-	P	P	P	P	P	P	P	P			Eburnamenine(13aR-cis)-610,470)	
**23.169**	2-Methyl-7-phenylindole												
**23.195**	Eburnamenin-14-ol, 14,15-dihydro-, (14.beta.)-			P								14-Isoeburnamine (619344)	
**23.218**	Quebrachamine	P	P	P	P	P	P	P	P	P			
**23.440**	l-Alanine, n-propargyloxycarbonyl-, ethyl ester	P	P		P	P			P				
**23.806**	2,4-Diamino-6-methyl-1,3,5-triazine												
**23.899**	Indolo[2,3-a]quinolizin-4(12H)-one, 1,2,3,6,7,12b-hexahydro-3,12b-dimethyl-			P									
**24.029**	Aspidospermidine, 1-ethyl-			P		P							
**24.165**	Eburnamenine-14-methanol, 14,15-dihydro-, (3.alpha.,14.alpha.,16.alpha.)-								P			Vincaminol (201188)	
**24.360**	Akuammilan-17-oic acid, methyl ester	P		P	P	P		P		P		Strictamine (5324377)	
**24.647**	2H-3,7-Methanoazacycloundecino[5,4-b]indole-9-carboxylic acid, 5-ethyl-1,4,5,6,7,8,9,10-octahydro-, methyl ester, [5S-(5R*,7R*,9S*)]-											Cleavamine (425980)	
**24.761**	Phthalic acid, 2-ethylbutyl nonyl ester			P									
**25.092**	Vinburnine	P	P	P	P	P			P			Eburnamonine (71203)	Antidiabetic [44]
**25.817**	Phthalic acid, bis(7-methyloctyl) ester			P				P					
**26.081**	Squalene			P		P	P	P	P	P			Antidiabetic [45] Antioxidant [46]
**26.328**	Eburnamenine-14-carboxylic acid, 14,15-dihydro-14-hydroxy-, methyl ester, (3.alpha.,14.alpha.,16.alpha.)-										P	Vincamine (15376)	Antidiabetic Activity [44]
**26.766**	Apovincamine		P	P			P						

## Discussion

*Rhazya stricta* is claimed to contain various medicinal properties including antihyperglycemic effects like some other members of Apocynaceae family e.g., *Telosma procumbens* Merr., *Vinca rosea* L., *Picralima nitida* [47]*.* Root extracts of the plant inhibited α-glucosidase enzyme that can lead to better glycemic control through a reduction in disaccharide hydrolysis [48]. The inhibition of the DPP-IV enzyme is another strategy to keep incretin hormones available in the blood for insulin-dependent disposal of glucose from the blood. Our study revealed that *R. stricta* root has dual activity to cope with DM as it inhibited the DPP-IV enzyme as well as stimulated GLP1 secretion. Despite the availability of many synthetic DPP-IV inhibitors, various studies have been conducted on medicinal plants and several plants are found to be active against DPP-IV including *Mangifera indica*, *Berberis aristata* (turmeric)*, Inonotus obliquus* (a mushroom), *Ocimum sanctum* and *Momordica charantia* [49].

*Rhazya stricta* root extracts remarkably inhibition β-Secretase in a dose-dependent manner that is supposed to be involved in diet-induced obesity and related complications including diabetes. Recently, a study by Meakin et al., (2018) reported that BACE1 is a regulator of insulin signaling and the amount of insulin receptor in the liver and during diabetes, the amount of insulin receptor is found to be reduced due to the degradation by the BACE1 enzyme. In this connection, they suggested that insulin signaling can be improved with the use of BACE1 inhibitors during diabetes [50].

Continuous monitoring of the blood glucose levels of diabetic patients and HBA1C levels are considered important factors in the treatment of DM. The root extracts of *R. stricta* considerably reduced the blood glucose level and HbA1c in accordance with previous reports where aerial parts of the plant were seen reducing blood glucose levels and HbA1c accordingly [13, 14]. Another study reported that long-term oral administration of alkaloids rich fractions of *R. stricta* significantly improved the insulin level and insulin resistance which showed insulin secretagogue activity of the plant for the disposal of blood glucose [16].

An array of secondary metabolites is well-known for the treatment of diabetes reported from plant species. Initial in vitro*,* in vivo and enzyme based assays were considered strong tools to assess the plants’ antidiabetic potentials. After the initial screening, we have found a variety of the compounds of different phytochemical classes through GCMS. Interestingly, among the identified compounds in the SCL fraction, five have already been documented as antihyperglycemic compounds namely; Hexadecane [40], n-Hexadecanoic acid [41], Vinburnine, Vincamine [44] and Squalene [45]. Some other classes of compounds have also been identified and derivatives of which are reported of antidiabetic potential like Benzofuran [51], Quinoxaline [52] Quinoline [53, 54], Pyridine [55], Sydnones [56] Thioureas [57] and Isoquinolines [58]. Moreover, Berberine, that is an Isoquinoline alkaloid isolated from *Rhizoma coptidis* has shown hypoglycemic and anti-dyslipidemic activities [58].

Several studies reported the link of reactive oxygen species with diabetes and oxidative stress is considered as one of the main factors that have an important role in beta-cells dysfunction, resulting in diabetes [59]. The role of oxidative stress has also been determined in the pathogenesis and complication of diabetes [60, 61]. In this regard, various reports suggested the use of antioxidants in the aetiology of diabetes and its associated complications [62, 63]. Up-till now, many plants have been investigated extensively to search natural antioxidants to prevent/minimize the destructive process caused by the oxidative stress [64]. Several natural products are reported here which have proven antioxidant attributes including thioureas derivatives [57] trans-13-Octadecenoic acid, methyl ester, 9-Octadecenoic acid (Z)-, methyl ester [42], uleine [43], acetamide derivatives [65] and squalene [46]. Thus, the *Rhazya stricta* root extracts displayed its potential for DM by lowering blood glucose level through different mechanisms that might be a synergistic effect of many of the phytoconstituents present in the extracts.

In this study, positive effects of the extracts on other biochemical parameters like lipid profile, renal function tests, liver function tests, total protein and serum albumin of mice have also been observed. The outcome of these biochemical parameters pointed out lipid-lowering potential and beneficial effects of the root extract on kidneys and liver functions of the mice. The extract of the leaves of *R. stricta* was reported in lowering the levels of cholesterol and triglycerides in animals [13, 14, 16, 66]. The histological examination of the mice liver has shown that the administration of the root extract fractions has not posed any substantial alterations of liver cells and any kind of known hepatic variations like dilation, congestion, necrosis or hypertrophy were not seen in all treated groups. These results suggested that the root extracts of *R. stricta* has potential to preserve the hepatic cells against diabetes led changes and its associated complaints.

Furthermore, the acceptance of findings of plant based natural products lead animal research for human is debatable but keeping in view a wide range of commonalities in both human and mice, the results of basic research are required to be tested on human considering that these secondary metabolites are safe. We suggest root extract fractions of *R. stricta* as a strong candidate possessing antidiabetic activity and these results shows that our methodology achieved the main objectives of the study. The main limitation of using mice in this kind of research is the “blood volume” as effects of plant extracts on many other blood parameters could be done. However, number of animals can be reduced for this kind of basic research which may have no or limited impacts on outcome.

## Conclusion

The root part of *R. stricta* is a potential source for the treatment of DM as it has demonstrated its effectiveness by inhibiting various key enzymes involved in the hyperglycemia. All the conducted assays clearly indicated the role of *R. stricta* root in lowering of blood glucose levels with a positive impact on the associated biochemical parameters and liver cells. Moreover, a variety of natural products including alkaloids, heterocyclic compounds etc. are identified in the root extract of *R. stricta*. We suggest a possible synergistic involvement of these identified phytochemicals in the antidiabetic activity of the extract.

## Data Availability

All data/material is available on request from the corresponding author.

## Abbreviations

ALT: Alanine Transaminase
AST: Aspartate Transaminase
ALP: Alkaline Phosphatase
BACE-1: Beta-amyloid (APP) cleaving enzyme
DM: Diabetes Mellitus
DPP-IV: Dipeptidyl peptidase-IV
DMEM: Dulbecco’s Modified Eagle Medium
FAL: First Aqueous Layer
FCL: Forth Chloroform Layer
FCC: Flash column chromatography
GLP-1: Glucagon-like peptide-1
HbA1c: Glycosylated hemoglobin
GC-MS: Gas Chromatography-Mass Spectrometry
SCL: Second Chloroform Layer
TAL: Third Aqueous Layer
TLC: Thin Layer Chromatography
